# Limited Accuracy of Tibial Stylus Measurements in Conventional Total Knee Arthroplasty

**DOI:** 10.3390/jcm15103935

**Published:** 2026-05-20

**Authors:** Oliver Haider, Tobias Scheidl, Anna Jungwirth-Weinberger, Amrut Diwakar Raje, Darshan S. Angadi, Zoltan Buday, Katharina Muellner, Maximilian F. Kasparek, Thomas Muellner

**Affiliations:** 1 Vienna General Hospital, Medical University of Vienna, Waehringer Guertel 18–20, 1090 Vienna, Austria; 2 Department of Orthopedic Surgery and Traumatology, Evangelisches Krankenhaus Wien, Hans-Sachs-Gasse 10–12, 1180 Vienna, Austria; 3 Department of Orthopaedics, Wockhardt Hospital, Mira Road, Mumbai 400011, India; 4 Department of Orthopaedic Surgery and Traumatology, Moventris Health Sciences, C103 Renaissance Exotica, Bengaluru 560001, India; 5 Department of Orthopedic Surgery and Traumatology, Klinik Donaustadt, Langobardenstraße 122, 1220 Vienna, Austria

**Keywords:** total knee arthroplasty, tibial resection, jig accuracy, stylus measurement, bone conservation

## Abstract

**Background**: Accurate tibial resection is important for component alignment and implant longevity in total knee arthroplasty (TKA). Despite the utilization of standardized instrumentation, discrepancies between predicted and actual resection depth persist. In the current study, the correlation between stylus-derived measurements and actual tibial resection thickness is analyzed. **Methods**: A total of 109 patients undergoing primary TKA using the Legion system (Smith & Nephew, Memphis, TN, USA) were included. The intraoperative resection depth was measured with a stylus at the selected tibial reference point and compared with the caliper-measured osteochondral thickness of the resected tibial fragment at the same location. Correlations were assessed using Spearman’s rank correlation, while agreement was evaluated using Bland–Altman analysis. **Results**: The mean stylus-indicated resection depth was 4.24 ± 2.15 mm, whereas the mean caliper-measured osteochondral thickness at the same marked reference point was 5.75 ± 2.30 mm. After adjustment for the 1.37 mm saw blade kerf, the mean osteochondral resection thickness was 7.12 ± 2.30 mm. The mean discrepancy between planned and kerf-adjusted osteochondral resection was −2.85 (SD = 1.99 mm; 95% CI: −3.22 to −2.47 mm) indicating a tendency toward over-resection. The correlation between stylus-indicated depth and actual osteochondral resection thickness was moderate (ρ = 0.540, *p* < 0.001). Bland–Altman analysis showed wide 95% limits of agreement from −6.75 to 1.05 mm. **Conclusions:** Stylus-based predictions of tibial resection depth show limited accuracy. Stylus-based measurements should be interpreted with caution and complemented by intraoperative verification.

## 1. Introduction

Total knee arthroplasty (TKA) represents the prevailing gold standard for cases of advanced knee osteoarthritis, offering patients substantial pain relief and functional improvement, when conservative measures have proven unsuccessful [1,2]. Optimal implant positioning and joint line restoration are essential for long-term implant survivorship and patient satisfaction [3]. Among all technical steps, the accuracy of tibial bone resection plays a critical role in ensuring proper alignment, balanced flexion–extension gaps, and avoiding early component failure [4,5].

The majority of contemporary TKA systems rely on an extramedullary tibial alignment guide in conjunction with a stylus to ascertain the optimal tibial resection depth. The stylus is attached to the tibial cutting guide and set to a predetermined depth. The surgeon places the stylus tip on a selected reference point. The cutting block is then positioned so that the planned resection will remove the desired thickness of bone relative to this point, typically compensating for cartilage wear. Despite the standardized use of conventional instrumentation, previous studies have reported inaccuracies in tibial component alignment and positioning when conventional tibial cutting guides are used, potentially leading to tibial over-resection [6,7]. Increased tibial resection depth has been associated with greater valgus laxity in flexion [8]. Moreover, deeper tibial resections may alter proximal tibial strain distribution and increase metaphyseal loading, which could affect tibial fixation [4]. From a clinical perspective, tibial resection inaccuracies may also influence polyethylene insert selection. Thicker inserts have been associated with altered ligament forces, increased tibiofemoral compressive forces, and a higher failure rates in primary TKA [9,10]. Additionally, inaccurate tibial resection may contribute to alterations in joint-line position, particularly when excessive tibial bone removal requires compensation with thicker polyethylene inserts to restore gap balance. Impaired joint-line restoration has been associated with inferior functional outcomes after primary TKA [11] and increased polyethylene wear, especially when combined with changes in posterior tibial slope [12].

Recent technological advancements, including computer-assisted navigation and robotic-assisted TKA, have demonstrated enhanced precision in component positioning and bone resections, resulting in a reduced variability in tibial cut depth [13,14,15]. However, the accessibility of these systems varies due to regional discrepancies, and conventional jig-based techniques prevail as the primary method for measuring tibial resection depth [16,17,18].

Despite the extensive utilization of the tibial stylus, a limited number of studies have directly quantified the correlation between stylus readings and actual tibial resection thickness. Recognizing the potential clinical consequences of even minor deviations, the objective of the current study was to evaluate the accuracy of stylus-based tibial resection in primary TKA. Specifically, we sought to assess the association and agreement between intraoperatively recorded stylus measurements and the actual osteochondral resection thickness measured with a caliper. An enhanced understanding of the inherent limitations of manual instrumentation, particularly in settings without access to technology-assisted surgery, may assist surgeons in implementing intraoperative modifications, thereby preventing unnecessary bone loss. We hypothesized that stylus-derived tibial resection measurements would show only limited agreement with the kerf-adjusted actual resection thickness.

## 2. Materials and Methods

This retrospective single-center observational cohort study was conducted between 2017 and 2019 and included 115 consecutive patients who underwent primary TKA using the LEGION^®^ Total Knee System (Smith & Nephew, Memphis, TN, USA). The inclusion criteria encompassed symptomatic, end-stage primary osteoarthritis and scheduled unilateral TKA utilizing the Legion knee system. Exclusion criteria were prior revision arthroplasty, secondary osteoarthritis due to trauma or infection, severe tibial bone loss, or deformities that precluded standard instrumentation.

Of the initially included 115 patients, six cases were excluded because an additional tibial recut was required after the initial resection. These cases were excluded because the second resection resulted in only a thin additional bone wafer, which could not be reliably measured with the caliper and would not have allowed a valid comparison between the stylus-indicated planned resection depth and the actual resected osteochondral thickness. Therefore, the final study cohort consisted of 109 patients. Prior to the study, ethical approval was obtained from the local institutional review board (#01/2023).

The decision to exclusively include patients treated with the LEGION^®^ Total Knee System (Smith & Nephew, Memphis, TN, USA) was made due to its predominant usage during the specified period and to avoid potential deviations when comparing tibial resections performed with different instrumentation.

Preoperative planning was conducted in accordance with standard protocol, which included long-leg standing radiographs to assess coronal alignment and deformity type. Preoperative templating was performed to guide implant selection and resection planning. All surgical procedures were performed under standardized conditions by a high-volume arthroplasty surgeon (TM). The surgical approach was determined by the surgeon’s professional judgment and was either a medial parapatellar or a midvastus approach. The tibial resection was performed using an extramedullary alignment guide and a conventional stylus to determine the resection depth.

The extramedullary tibial alignment guide and stylus system provided an intraoperative estimation of the planned resection depth. The stylus-indicated resection depth (in mm) was recorded intraoperatively prior to performing the tibial cut. A standardized oscillating saw blade with dimensions of 18 mm width, 90 mm length, and 1.37 mm thickness was used for all resections.

The stylus reference point was selected according to the surgeon’s intraoperative judgment and could be located either on the medial or lateral tibial plateau, depending on the individual pattern of cartilage wear and deformity.

The resected osteochondral fragment of the tibia was measured using a calibrated digital caliper (Smith & Nephew, Memphis, TN, USA) on the same point on the tibial plateau where the stylus was positioned. Measurements were performed on the resected specimens as removed, including any residual articular cartilage attached to the subchondral bone surface.

**Figure 1:**
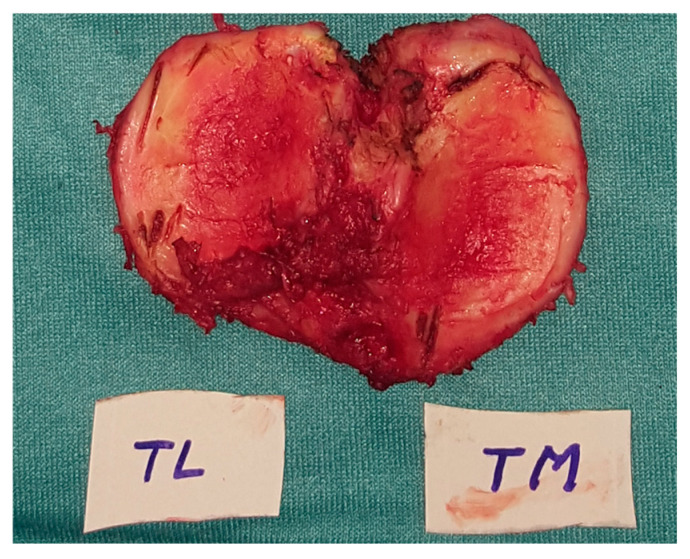
Example of tibial resection after using the stylus for measuring the resection depth. TL—Lateral tibial Cut, TM—Medial tibial cut.

**Figure 2:**
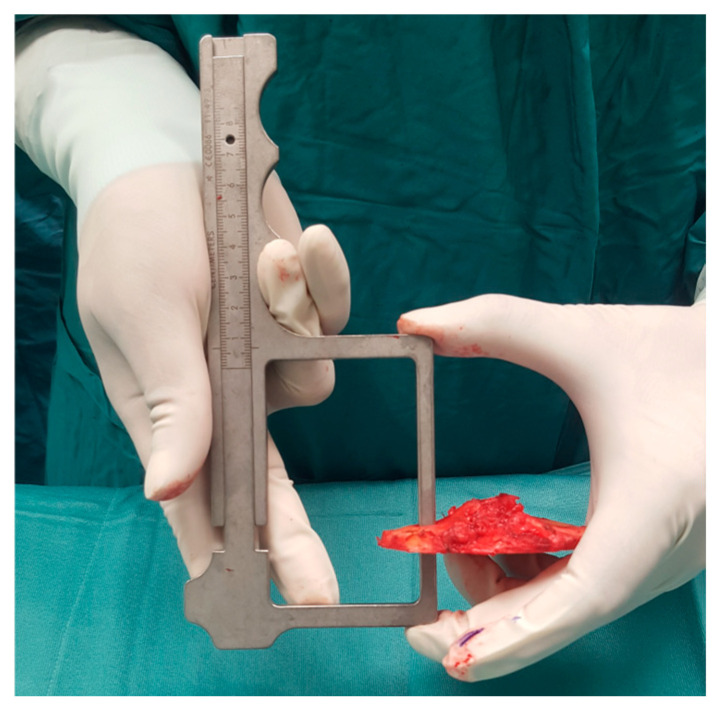
Measurement of the osteochondral thickness of the resected tibial fragment using a calibrated digital caliper.

Therefore, the recorded values represent the total resected osteochondral thickness rather than the thickness of subchondral bone alone. Measurements were always performed by the same surgeon (TM) at the same location and documented intraoperatively. All resections were performed using the same implant system, conventional tibial alignment guide, oscillating saw blade thickness, and measurement protocol. Figure 1 and Figure 2 show the resected tibial plateau and its measurement with the caliper.

The error was defined as stylus-indicated planned resection depth minus osteochondral resection thickness; therefore, negative values indicate over-resection, whereas positive values indicate under-resection. The primary error definition was based on the kerf-adjusted osteochondral resection thickness, calculated as the caliper-measured osteochondral thickness plus the 1.37 mm saw blade thickness. This approach was chosen because the saw blade kerf represents a physical component of the total tissue removed during tibial resection. The primary accuracy assessment was therefore based on the discrepancy between the stylus-indicated planned resection depth and the kerf-adjusted osteochondral resection thickness. For transparency, unadjusted error was additionally calculated using the caliper-measured osteochondral thickness without adding the saw blade thickness.

The distribution of all variables was assessed using the Shapiro–Wilk test. For descriptive purposes, variables were presented as means with standard deviations. The association between stylus-indicated resection depth and kerf-adjusted osteochondral resection thickness was assessed using Spearman’s rank correlation coefficient (ρ). Since the kerf adjustment consisted of adding a constant value to all measured resections, the rank order of measurements and therefore Spearman’s ρ was not influenced by this adjustment.

Agreement between stylus-indicated planned resection depth and kerf-adjusted osteochondral resection thickness was assessed using Bland–Altman analysis. The difference was calculated as stylus-indicated resection depth minus kerf-adjusted osteochondral resection thickness; therefore, negative values indicate over-resection. The mean difference and 95% limits of agreement were calculated. In addition, linear regression analysis of the difference against the mean of both measurements was performed to assess potential proportional bias, and the proportions of cases with absolute discrepancies exceeding 2 mm and 3 mm were calculated. A *p*-value of < 0.05 was considered statistically significant, while a *p*-value of < 0.001 was deemed as highly significant. The data was analyzed using IBM SPSS Statistics, version 30.0.0.0 (IBM, Armonk, NY, USA). The mediCAD^®^ software 7.0 (mediCAD Hectec GmbH, Altdorf/Landshut, Germany) was used for all preoperative radiographic measurements.

## 3. Results

The study cohort comprised 109 patients, including 65 women (59.6%) and 44 men (40.4%), with a mean age of 72.5 ± 8.55 years. Varus deformity was present in 70 patients (64.2%). A total of 57 right knees (52.3%) and 52 left knees (47.7%) underwent surgery. All patients were treated with the LEGION^®^ Total Knee System (Smith & Nephew, Memphis, TN, USA). Of the 109 TKAs, 32 (29.4%) were performed using a cruciate-retaining (CR) design and 77 (70.6%) using a posterior-stabilized (PS) design. Based on the surgeon’s intraoperative judgment, the stylus reference point was located on the medial tibial plateau in 83 cases (76.1%) and on the lateral tibial plateau in 26 cases (23.9%).

Across all patients, the mean stylus-indicated resection depth at the selected intraoperative reference point was 4.24 ± 2.15 mm. Subsequent caliper measurement of the resected osteochondral tibial fragment at the same marked reference point showed that the measured osteochondral resection thickness exceeded the corresponding stylus-indicated value in most cases. The mean caliper-measured osteochondral thickness was 5.75 ± 2.30 mm. After adjustment for the 1.37 mm saw blade kerf, the mean kerf-adjusted osteochondral resection thickness was 7.12 ± 2.30 mm.

At the marked stylus reference point, the mean difference between the stylus-indicated planned resection depth and the caliper-measured osteochondral thickness was −1.48 ± 1.98 mm. After adjustment for the 1.37 mm saw blade kerf, the mean discrepancy between the stylus-indicated planned resection depth and the kerf-adjusted osteochondral resection thickness was −2.85 ± 1.99 mm.

Osteochondral resection thickness was further analyzed according to the location of the selected stylus reference point. For medial reference point placement, the mean stylus-indicated resection depth was 3.87 ± 1.72 mm, the mean caliper-measured osteochondral thickness was 5.21 ± 1.89 mm, and the mean kerf-adjusted osteochondral resection thickness was 6.57 ± 1.89 mm. For lateral reference point placement, the corresponding values were 5.42 ± 2.89 mm, 7.50 ± 2.65 mm, and 8.87 ± 2.65 mm, respectively. The mean discrepancy between the stylus-indicated planned resection depth and the kerf-adjusted osteochondral resection thickness was −2.66 ± 2.01 mm for medial reference point placement and −3.45 ± 1.80 mm for lateral reference point placement.

Table 1 summarizes the mean caliper-measured osteochondral thickness and the mean error between the stylus-indicated planned resection depth and the osteochondral resection thickness, both with and without adjustment for the 1.37 mm saw blade kerf.

The Spearman correlation coefficient between the stylus-indicated planned resection depth and the kerf-adjusted osteochondral resection thickness measured at the same marked reference point was ρ = 0.540 (*p* < 0.001). Since kerf adjustment consisted of adding a constant value to all caliper-measured resections, Spearman’s ρ remained unchanged compared with the unadjusted analysis.

Bland–Altman analysis was performed to assess agreement between the stylus-indicated planned resection depth and the kerf-adjusted osteochondral resection thickness. The mean difference was −2.85 mm, indicating that the osteochondral resection thickness exceeded the stylus-indicated planned depth by 2.85 mm on average. The 95% limits of agreement ranged from −6.75 to 1.05 mm, demonstrating considerable variability between both measurements. There was no significant association between the difference and the mean of both measurements (*p* = 0.398), suggesting the absence of proportional bias.

When accounting for the 1.37 mm saw blade kerf, an absolute discrepancy greater than 2 mm was observed in 77 of 109 cases (70.64%), and an absolute discrepancy greater than 3 mm in 52 of 109 cases (47.71%). Without this adjustment, the corresponding values were 27 of 109 cases (24.77%) and 16 of 109 cases (14.68%), respectively.

Despite the moderate correlation, the Bland–Altman analysis and categorical error assessment demonstrated considerable disagreement between stylus-indicated planned resection depth and kerf-adjusted osteochondral resection thickness. When accounting for saw blade kerf, concordance between stylus-indicated and actual resection thickness was observed in 9 knees (8.26%), whereas 98 knees (89.91%) showed over-resection and 2 knees (1.83%) under-resection. In the unadjusted analysis, concordance was present in 20 knees (18.3%), while over-resection and under-resection were observed in 78 (71.6%) and 11 knees (10.1%), respectively.

**Figure 3:**
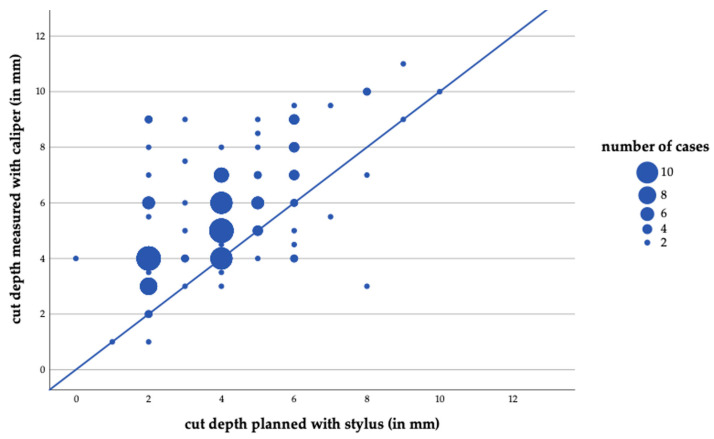
Scatter plot showing the relationship between stylus-indicated planned resection depth and the unadjusted osteochondral resection thickness. The solid diagonal line indicates concordant values.

The relationship between the stylus-indicated planned resection depth and the unadjusted osteochondral resection thickness is illustrated in Figure 3. The solid diagonal line represents perfect concordance between both measurements. Data points located above this line indicate over-resection, meaning that the actual resection thickness exceeded the stylus-indicated planned depth, whereas points below the line indicate under-resection.

To determine whether the correlation between planned and kerf-adjusted osteochondral resection was stronger in a specific subgroup, Spearman’s correlation analysis was conducted separately for medial and lateral reference point placement. To further explore whether preoperative deformity type influenced the association between stylus-indicated planned resection depth and kerf-adjusted actual resection thickness, an additional subgroup analysis was performed for varus and valgus knees. The Spearman correlation coefficient was ρ = 0.593 (*p* < 0.001) in varus knees and ρ = 0.324 (*p* = 0.044) in valgus knees. The weaker correlation observed in valgus cases should be interpreted cautiously because of the smaller subgroup size and the distinct anatomical characteristics of valgus knees. The results are summarized in Table 2.

The inaccuracy of the stylus-based measurement is further reflected in the distribution of polyethylene insert thicknesses. The LEGION^®^ Total Knee System (Smith & Nephew, Memphis, TN, USA) is conventionally designed for a standard 9 mm insert. In the present cohort, however, a standard 9 mm insert was implanted in 59 of 109 cases (54.1%), whereas 50 knees (45.9%) received inserts thicker than 9 mm. Detailed information on the distribution of insert thicknesses is provided in Table 3.

## 4. Discussion

The present study evaluated the association and agreement between the planned tibial resection depth, as determined by a stylus on the tibial cutting guide, and the kerf-adjusted osteochondral resection thickness. The most notable finding was that actual kerf-adjusted resection thickness exceeded the stylus-indicated planned resection depth in most cases. These findings indicate a substantial discrepancy between the planned and osteochondral tibial resections, which is also underlined by the moderate correlation between the two (ρ = 0.540, *p* < 0.001).

Despite the fact that jigs and guides were primarily implemented with the intention of standardizing resections, our findings emphasize persistent concerns regarding the precision of conventional extramedullary alignment systems. Chiu et al. [19] and Iorio et al. [7] focused on the accuracy of the tibial alignment in the coronal plane, and their findings revealed that manual tibial guide systems provided unsatisfactory results with regard to tibial component placement. Chiu et al. [19] reported that inconsistent placement of the tibial alignment guide can lead to variability in tibial component positioning, even when using standard instruments. It was recommended that additional visual cues and palpation be used during the placement of the extramedullary alignment guide, with a purpose to increase precision and avoid inaccurate tibial bone cuts. Furthermore, Iorio et al. [7] highlighted the inherent limitations of manual tibial guide systems, which are attributed to both intraoperative and anatomic factors that compromise accuracy. They observed a tendency towards varus malalignment and a decreased tibial slope, when using conventional instrumentation for tibial resection. The results of the present study serve to further emphasize these findings, indicating that stylus-based depth measurement in isolation may not ensure adequate control over resection accuracy. This is not only evident in the precise alignment of the tibial component in the coronal plane, but also regarding the tibial resection depth.

However, Leal-Blanquet et al. [6] reported only a modest discrepancy between planned and achieved coronal bone-cut orientation when conventional cutting guides were used, with a mean absolute error of 1.31° (SD ± 2.54) for the tibial coronal cut, and concluded that this deviation was probably too small to meaningfully affect clinical outcomes. However, their analysis was based on postoperative radiographic coronal alignment and therefore addressed a different endpoint than the present study. In a more recent study, Cho et al. [20] demonstrated that the coronal accuracy of extramedullary tibial alignment can be improved by supplementing conventional instrumentation with preoperative radiographic references and intraoperative C-arm-guided verification, suggesting that additional reference methods may improve the reproducibility of conventional extramedullary systems.

A notable aspect of our cohort was the high proportion of knees with varus deformity. In the exploratory deformity-based subgroup analysis, the correlation between stylus-indicated planned resection depth and kerf-adjusted osteochondral resection thickness was stronger in varus knees than in valgus knees. However, the weaker correlation observed in valgus cases should be interpreted cautiously, as it may reflect both the smaller subgroup size and valgus-specific anatomical variability rather than a definitive difference in stylus accuracy between deformity phenotypes. Valgus knees may present distinct characteristics, including lateral compartment wear, lateral tibial plateau hypoplasia, and greater variability in asymmetric resection requirements, which may affect both stylus reference point selection and resection reproducibility. Schnurr et al. [21] demonstrated that deformity type, particularly valgus alignment, can influence tibial resection requirements, often necessitating smaller or more asymmetric resections than in varus knees. Importantly, deformity type and stylus reference point placement were not identical in all cases; therefore, both deformity phenotype and actual reference point location should be considered when interpreting subgroup findings. Overall, the variability observed in resection depth relative to stylus input highlights the limitations of a one-size-fits-all approach.

From a clinical perspective, these findings suggest that surgeons should not rely exclusively on stylus measurements when determining resection depth. This is further supported by the Bland–Altman analysis: although no significant proportional bias was observed, the systematic bias and wide limits of agreement indicate that stylus-based measurements remain insufficiently precise for reliable prediction of the osteochondral resection thickness in individual cases. Stylus-based systems assume a uniform reference point, but they do not account for variations in cartilage loss, bone erosion, or subchondral sclerosis, all of which may affect both the level and quality of the tibial cut. Given that differences of only a few millimeters may be clinically relevant in total knee arthroplasty, the observed variability may have implications for bone preservation, soft-tissue balance, and restoration of the joint line, although these clinical consequences could not be directly assessed in the present study. To enhance precision in TKA, technological solutions such as computer-assisted navigation and robotic-assisted systems are increasingly being adopted. These tools provide real-time feedback and enable more consistent resection depths and component alignment. Their use has been associated with improved implant positioning and reduced variability in bone resection and component alignment [14,15,16]. While these technologies are not yet universally available, their integration into routine practice could address many of the challenges associated with traditional instrumentation. Furthermore, ongoing innovations in jig design—such as adjustable resection blocks or integrated depth sensors—may offer more accessible alternatives to enhance intraoperative control. However, because conventional jig-based techniques remain widely used, stylus-based measurements should not be relied on in isolation. Instead, the planned tibial resection should be cross-checked intraoperatively by direct measurement of the resected osteochondral thickness at the marked stylus reference point, combined with verification against anatomical landmarks and assessment of flexion–extension gap balance. In our cohort, actual resection thickness frequently exceeded the stylus-indicated value, indicating a tendency toward over-resection. Awareness of this pattern may help surgeons critically reassess the initial tibial cut and avoid unnecessary bone loss before proceeding with implant and polyethylene insert selection.

The use of inserts thicker than the standard 9 mm may be clinically relevant, as increased polyethylene thickness can influence soft-tissue tension, gap balance, and the reconstructed joint line. However, postoperative joint-line height was not systematically assessed; therefore, it remains unclear whether thicker inserts resulted in unintended joint-line elevation. Since insert selection is multifactorial, the observed insert distribution should be interpreted as an indirect marker of the potential clinical relevance of resection depth rather than as direct evidence of joint-line elevation.

This study has several limitations. Bone density was not measured and subchondral sclerosis at the stylus reference site was not systematically graded or documented intraoperatively. Although local bone quality was visually and tactilely assessed by the surgeon as part of routine surgical judgment, this assessment was not recorded in a standardized but was determined intraoperatively by the surgeon according to the individual pattern of cartilage wear and deformity. Although this reflects routine clinical practice, it may limit reproducibility. Furthermore, caliper measurements were performed on the resected osteochondral specimens as removed, including any residual articular cartilage. Although measurements were obtained at the same location where the stylus had previously been positioned, local variations in residual cartilage thickness may have influenced the recorded values, as cartilage thickness at the specific measurement site was not quantified. Resection thickness was measured only at the stylus reference point and not systematically at multiple anterior, posterior, medial, or lateral locations. Therefore, the present study cannot distinguish between a uniform vertical shift and an angular deviation of the tibial cut, for example due to blade deflection or cutting-guide positioning. In addition, patients requiring an additional tibial recut were excluded because the secondary bone wafer could not be reliably measured. This may have introduced selection bias, as these cases may have represented larger deviations after the initial tibial cut, potentially leading to underestimation of the true inaccuracy of stylus-guided tibial resection. Moreover, the degree of osteoarthritis was not systematically graded using the Kellgren–Lawrence classification or another standardized cartilage grading system. Since both the stylus reading and the caliper measurement were obtained at the same marked anatomical reference point, a global radiographic grading of osteoarthritis was considered to have limited additional value for the present analysis. Kellgren–Lawrence grading reflects the overall radiographic severity of osteoarthritis but does not quantify the exact residual cartilage thickness at the specific intraoperative point of stylus placement. Therefore, it would not have allowed reliable adjustment for local cartilage thickness at the measurement site. Although kerf adjustment was performed by adding the constant 1.37 mm saw blade thickness, the actual local effect of the saw blade may vary depending on blade position within the cutting slot, blade deflection, bone hardness, and cutting-guide stability. Therefore, the kerf-adjusted value should be interpreted as an approximation of total tissue removal rather than an exact measurement of the local saw path. Accordingly, unadjusted values based on caliper-measured osteochondral thickness alone were also reported for transparency. Because of the retrospective design of the present study, postoperative functional outcomes and their relationship to resection accuracy could not be evaluated. Future studies incorporating these factors will be important to allow a more comprehensive assessment of this issue and to better clarify its potential clinical implications. Finally, postoperative joint-line height and patellofemoral kinematics were not systematically assessed. Therefore, the present study cannot determine whether the use of thicker polyethylene inserts resulted in unintended joint-line elevation or clinically relevant changes in patellofemoral mechanics.

## 5. Conclusions

The present study demonstrates limited agreement between stylus-indicated planned resection depth and kerf-adjusted osteochondral tibial resection thickness in conventional TKA. Although a moderate correlation was observed, Bland–Altman analysis showed systematic over-resection and wide limits of agreement, indicating that stylus-based measurements should be interpreted with caution.

## Figures and Tables

**Table 1:** Mean values for caliper-measured osteochondral thickness and error between the stylus-indicated planned resection depth and the actual resection thickness with and without adjustment for saw blade kerf.

	Mean Caliper-Measured Osteochondral Thickness		Mean Error	
	Without Kerf	With Kerf	Without Kerf	With Kerf
**All cases** **(n = 109)**	5.75 ± 2.30 mm	7.12 ± 2.30 mm	−1.48 ± 1.98 mm	−2.85 ± 1.99 mm
**Reference point medial** **(n = 83)**	5.21 ± 1.89 mm	6.57 ± 1.89 mm	−1.29 ± 2.01 mm	−2.66 ± 2.01 mm
**Reference point lateral** **(n = 26)**	7.50 ± 2.65 mm	8.87 ± 2.65 mm	−2.08 ± 1.80 mm	−3.45 ± 1.80 mm

**Table 2:** Spearman’s ρ for different subgroups.

Subgroup	n	ρ	*p*
Reference point medial	83	0.445	<0.001
Reference point lateral	26	0.721	<0.001
Varus	70	0.593	<0.001
Valgus	39	0.324	0.044

**Table 3:** Frequency of used insert thickness.

Insert Thickness (in mm)	Frequency
9	59
10	4
11	31
12	2
13	10
15	2
18	1

## Data Availability

The original contributions presented in this study are included in the article. Further inquiries can be directed to the corresponding author.

## References

[1] Carr A.J., Robertsson O., Graves S., Price A.J., Arden N.K., Judge A., Beard D.J. (2012). Knee replacement. Lancet.

[2] Price A.J., Alvand A., Troelsen A., Katz J.N., Hooper G., Gray A., Carr A., Beard D. (2018). Knee replacement. Lancet.

[3] Ritter M.A., Davis K.E., Meding J.B., Pierson J.L., Berend M.E., Malinzak R.A. (2011). The effect of alignment and BMI on failure of total knee replacement. J. Bone Jt. Surg. Am..

[4] Berend M.E., Small S.R., Ritter M.A., Buckley C.A. (2010). The effects of bone resection depth and malalignment on strain in the proximal tibia after total knee arthroplasty. J. Arthroplast..

[5] Berend M.E., Ritter M.A., Meding J.B., Faris P.M., Keating E.M., Redelman R., Faris G.W., Davis K.E. (2004). Tibial component failure mechanisms in total knee arthroplasty. Clin. Orthop. Relat. Res..

[6] Leal-Blanquet J., Hinarejos P., Gimenez-Valero E., Torres-Claramunt R., Sánchez-Soler J., Erquicia J., Gil-González S., Zumel-Marne A., Monllau J.C. (2023). Bone cut accuracy in total knee arthroplasty: Do conventional cutting guides stay true to the planned coronal orientation of the components?. Appl. Sci..

[7] Iorio R., Bolle G., Conteduca F., Valeo L., Conteduca J., Mazza D., Ferretti A. (2013). Accuracy of manual instrumentation of tibial cutting guide in total knee arthroplasty. Knee Surg. Sports Traumatol. Arthrosc..

[8] Sappey-Marinier E., White N., Gaillard R., Cheze L., Servien E., Neyret P., Lustig S. (2019). Increased valgus laxity in flexion with greater tibial resection depth following total knee arthroplasty. Knee Surg. Sports Traumatol. Arthrosc..

[9] Tzanetis P., Marra M.A., Fluit R., Koopman H.F.J.M., Verdonschot N.J.J. (2021). Biomechanical consequences of tibial insert thickness after total knee arthroplasty: A musculoskeletal simulation study. Appl. Sci..

[10] Berend M.E., Davis P.J., Ritter M.A., Keating E.M., Faris P.M., Meding J.B., Malinzak R.A. (2010). “Thicker” polyethylene bearings are associated with higher failure rates in primary total knee arthroplasty. J. Arthroplast..

[11] Koshire S., Mohanty S.S., Keny S.A., Rai A.K., Rathod T.N., Kamble P. (2022). The influence of joint line restoration on functional outcome after primary total knee arthroplasty: A prospective study. J. Clin. Orthop. Trauma.

[12] Pourzal R., Theissmann R., Morlock M.M., Fischer A., von Knoch M., Grupp T.M. (2020). Joint line elevation and tibial slope are associated with increased polyethylene wear in cruciate-retaining total knee replacement. J. Orthop. Res..

[13] Wong W.K., Abu Bakar Sajak A., Chua H.S. (2024). Real-world accuracy of robotic-assisted total knee arthroplasty and its impact on expedited recovery. J. Robot. Surg..

[14] Yi J., Gao Z., Huang Y., Liu Y., Zhang Y., Chai W. (2024). Evaluating the accuracy of a new robotically assisted system in cadaveric total knee arthroplasty procedures. J. Orthop. Surg. Res..

[15] Gamie Z., Kenanidis E., Douvlis G., Milonakis N., Maslaris A., Tsiridis E. (2024). Accuracy of the imageless mode of the ROSA robotic system for targeted resection thickness in total knee arthroplasty: A prospective, single surgeon case-series study. Int. J. Med. Robot..

[16] Peterman N.J., Pagani N., Mann R., Li R.L., Gasienica J., Naik A., Sun D. (2024). Disparities in access to robotic knee arthroplasty: A geospatial analysis. J. Arthroplast..

[17] Inabathula A., Semerdzhiev D.I., Srinivasan A., Amirouche F., Puri L., Piponov H. (2024). Robots on the stage: A snapshot of the American robotic total knee arthroplasty market. JB JS Open Access.

[18] Jevnikar B.E., Khan S.T., Emara A.K., Elmenawi K.A., Deren M., Piuzzi N.S. (2025). Robotic total hip and knee arthroplasty: Economic impact and workflow efficiency. J. Robot. Surg..

[19] Chiu K.Y., Yau W.P., Ng T.P., Tang W.M. (2008). The accuracy of extramedullary guides for tibial component placement in total knee arthroplasty. Int. Orthop..

[20] Cho J.H., Choi J.Y., Lee S.S. (2022). Accuracy of the tibial component alignment by extramedullary system using simple radiographic references in total knee arthroplasty. Medicina.

[21] Schnurr C., Csécsei G., Nessler J., Eysel P., König D.P. (2011). How much tibial resection is required in total knee arthroplasty?. Int. Orthop..

